# MT-12 inhibits the proliferation of bladder cells *in vitro* and *in vivo* by enhancing autophagy through mitochondrial dysfunction

**DOI:** 10.1515/biol-2022-0082

**Published:** 2022-07-08

**Authors:** Yan Chen, Chengxing Xia, Chunwei Ye, Feineng Liu, Yitian Ou, Ruping Yan, Haifeng Wang, Delin Yang

**Affiliations:** Department of Urology, The Second Affiliated Hospital of Kunming Medical University, 374 Burma Avenue, Kunming 650101, China

**Keywords:** Cobra venom membrane toxin 12, bladder cancer, autophagy, mitochondrial dysfunction, ROS

## Abstract

Bladder cancer (BC) is one of the most common malignancies involving the urinary system. Our previous study demonstrated that cobra venom membrane toxin 12 (MT-12) could effectively inhibit BC cell growth and metastasis and induce apoptosis. However, the specific molecular mechanism remains unknown. In this study, we explored whether MT-12 inhibits BC cell proliferation by inducing autophagy cell death through mitochondrial dysfunction. As a result, MT-12 inhibited proliferation and colony formation in RT4 and T24 cells. In the BC xenograft mouse model, autophagy inhibitor 3-MA alleviated the inhibitory effect of MT-12 on tumor growth. In addition, immunostaining revealed downregulated autophagy in MT-12-treated RT4 and T24 cells. We also found that MT-12 led to dysfunctional mitochondria with decreased mitochondrial membrane potential, mtDNA abundance, and increased ROS production, ultimately inducing autophagic apoptosis *via* the ROS/JNK/P53 pathway. MT-12 inhibits BC proliferation *in vitro* and *in vivo* by enhancing autophagy. MT-12 induces mitochondrial dysfunction and decreases autophagy, leading to increased ROS production, which in turn activates the JNK/p53 pathway, leading to BC apoptosis.

## 1 Introduction

Bladder cancer (BC) is the seventh most common malignancy in male patients worldwide; the estimated incidence in the United States in 2019 was approximately 80,470, with an estimated mortality of 17,670. In the last decade, the incidence and mortality rates have increased yearly [1]. Approximately 75% of newly diagnosed BC cases have non-muscle-invasive bladder cancer (NMIBC), with a 5-year survival rate surpassing 88% [2]. NMIBC is treated by transurethral resection of bladder tumor (TURBT) combined with intravesical chemotherapy and immunosuppressive agents. However, the recurrence rate of NMIBC is approximately 50–70%, with 10–30% of recurrent cases showing a higher grade [3]. The long-term use of immunosuppressive agents and chemotherapeutic drugs leads to many side effects, including chemical cystitis, bone marrow transplantation, liver and kidney function damage, and blood cell reduction, making it difficult for some patients to adhere to the treatment [4]. Therefore, it is urgent to develop better methods for preventing BC recurrence.

Membrane toxin (MT), one of the major toxic components in cobras, has both cardiotoxic and cytotoxic effects. Studies have found that MT has strong antitumor activity, inhibits the biological activities of breast cancer, pancreatic cancer, and leukemia *in vitro* [5,6,7], and reduces tumor size *in vivo* [8]. Chinese cobra membrane toxin (MT-12), which belongs to the three-finger toxin family, is refined from cured Chinese naja venom. It is a strong alkali polypeptide that contains a large number of hydrophobic (60–63) amino acid residues [9]. Our previous report revealed that MT-12 effectively inhibits BC cell growth and metastasis [10], but the underlying mechanism remains unknown.

Autophagy is one of the important types of programmed cell death (PCD) closely related to cell proliferation. Mitochondria are not only the energy and metabolism centers of eukaryotic cells but also the primary regulators of PCD and signal transduction. The main role of the mitochondria in mammalian cells is to synthesize ATP and the byproduct, reactive oxygen species (ROS) [11,12], through oxidative phosphorylation. When the mitochondria are damaged by stimulation, the mitochondrial permeability transition pore (MPTP) is opened, reducing the mitochondrial membrane potential (MMP) [10,13], which releases cytochrome C (cyt C) and activates caspase 9, ultimately activating the caspase cascade and initiating endogenous cell apoptosis. Meanwhile, after MPTP opening, ROS [11,12] accumulate in the mitochondria and induce autophagy [14]. Recently, an increasing number of studies have shown that autophagy, also called type II PCD, plays an essential role in antitumor activities (e.g., apoptosis) in colon cancer [15], prostate cancer [16], and osteosarcoma [17]. Moreover, cobra venom cardiotoxins interact with the outer mitochondrial membrane, drawing our imagination between MT-12 and autophagy in the MT-12 molecular mechanism.

In this study, the biological effects of MT-12 in the BC cell lines T24 and RT4 on autophagy were separately inhibited, and mitochondrial function changes were investigated to determine the exploration mechanism of the antitumor activity of MT-12. Downregulated autophagy of MT-12 in BC apoptosis may provide clinical targets for non-muscular-invasive BC.

## 2 Methods

### 2.1 Drugs

MT-12, purified from the venom of the Chinese cobra *Naja atra*, was a gift from the Laboratory of Animal Toxins, Kunming Institute of Zoology, Chinese Academy of Medical Sciences, China. MT-12 was dissolved in dimethyl sulfoxide (DMSO; Sigma-Aldrich; Merck KGaA, Darmstadt, Germany) at a stock concentration of 4 mM and stored at −20°C. The human BC cell lines (T24 and RT4 cells) were obtained from our institute (Yunnan Institute of Urology).

### 2.2 Animals

A total of 24 BALB/c nude mice (aged 5 weeks, 20 ± 2 g) were purchased from the Animal Center of Sun Yat-Sen University (Guangzhou, China, KMMU2018013). Mice were maintained under specific pathogen-free conditions with a 12/12 h light–dark cycle.


**Ethical approval:** The research related to animal use has been complied with all the relevant national regulations and institutional policies for the care and use of animals and were approved by the Institutional Animal Care and Use Committee of the Second Affiliated Hospital of Kunming Medical University.

### 2.3 Cell culture

Human BC cell lines (T24 and RT4 cells) were cultured in RPMI-1640 (Gibco; Thermo Fisher Scientific Inc., Waltham, MA, USA) supplemented with 10% fetal bovine serum (HyClone, Logan, UT, USA) in 25 cm^2^ cell culture flasks (Corning Inc., Corning, NY, USA) at 37°C with 5% CO_2_.

### 2.4 CCK-8 cell proliferation assay

The cells were added to a 96-well plate at 100 μL/well (2 × 10^4^ cells) and precultured in RPMI-1640 for 24 h. According to previous findings, the IC_50_ of MT-12 in BC cells was approximately 0.51–0.66 μg/mL [18]. RT4 and T24 cells were treated with MT-12 (0, 0.1, 0.25, 0.5, and 1.0 μg/mL) for 24 and 48 h, respectively. Cell proliferation was determined by the CCK-8 assay (10 μL of solution per well). A microplate reader (Olympus, Tokyo, Japan) was used to determine the absorbance at 450 nm.

### 2.5 Colony formation assay

Cells (1.5 × 10^3^ per well) were seeded into six-well plates and cultured for 14 days to form visible colonies. The colonies were fixed with 10% neutral buffered formalin solution (Sigma-Aldrich Corporation) and stained with 0.01% crystal violet. After rinsing three times, visible colonies with more than 50 individual cells were counted using an SZX7 stereomicroscope (Olympus, Tokyo, Japan).

### 2.6 Measurement of apoptosis by flow cytometry

An Annexin V-FITC/PI kit (Dojindo, Japan) was used to detect cell apoptosis according to the manufacturer’s instructions. For example, the 1× Annexin V binding solution was premixed in a cell-seeded plate to generate a cell suspension with a final density of 1 × 10^6^ cells per mL. The cells were washed three times with phosphate-buffered saline (PBS) and mixed with 100 µL of Annexin V binding buffer, followed by the addition of 5 L each of Annexin V and PI. After incubation for 10–15 min in the dark, Annexin V binding buffer was added to bring the total volume to 1 mL. The cells were then transferred into flow cytometry tubes and analyzed by a flow cytometer (Ex = 488 nm and Em = 530 nm).

### 2.7 Transmission electron microscopy

To analyze the induction of autophagy in MT-12-treated BC cells morphologically, ultrastructural analysis was performed by transmission electron microscopy (TEM). Cells were fixed with 2.5% glutaraldehyde overnight at 4°C and subsequently fixed with 1% OsO_4_–0.15 M Na cacodylate/HCl. Then, the samples were dehydrated in graded ethanol, polymerized, and analyzed by electron microscopy.

### 2.8 Fluorescence electron microscopy

After 24 h of GFP-LC3 transfer into BC cells, the cells were subcultured in six-well plates covered with cover slides to induce autophagy. The culture medium was removed, and the slides were carefully washed once with PBS. Then, 3% paraformaldehyde was added to each well, and the culture slide was shaken at room temperature for 20 min, washed with PBS three times, and rocked with 2 mL of PBS at room temperature for 10 min. The cover slides were fixed with the fixation solution. After staining at room temperature for 1 min, the fluorescent puncta were observed by fluorescence microscopy (COXEM, Korea).

### 2.9 Western blot test

Treated cells were washed with cold PBS and lysed with radioimmunoprecipitation assay buffer (Beyotime, China) supplemented with protease inhibitors to extract total protein. Protein concentration was determined by the bicinchoninic acid protein assay. After denaturation, the proteins were separated by SDS-polyacrylamide gel electrophoresis and transferred onto PVDF membranes. Nonspecific binding was blocked with 5% skimmed milk in TBST buffer for 2 h, followed by incubation with primary antibodies (all 1:1,000; Abcam, Cambridge, UK) at 4°C overnight and secondary antibodies (1:5,000; Beyotime Biotechnology, Shanghai, China) at room temperature for 2 h. The blots were visualized using ECL detection reagents.

The list of antibodies is as follows: LC3II (ab192890), p62 (ab207305), beclin-1 (EPR20473), ROS (ab108492), JNK (ab273417), p53 (ab26), caspase-3 (ab32351), caspase-8 (ab32125), b-actin (ab8227), AMPK (ab32047), p-AMPK (ab92701), mTOR(ab134903), p-mTOR(ab109268), ULK1(ab240916), and p-ULK1(ab133747).

### 2.10 Human BC Xenograft mouse model

Mice were maintained under specific pathogen-free conditions. After acclimation for 1 week, the mice were randomly divided into three groups (*n* = 8 in each group, including four females and four males): vehicle control group (T24 + 0.05% DMSO), test group 1 (T24 + MT-12), and test group 2 (T24 + MT-12 + 3-MA). In all, 100 μL cell suspension (1 × 10^7^ cells/mL) was injected unilaterally. After tumor formation, the drug (2 mg/kg 3MA, 1 mg/kg MT12) is injected every 3 days. During the injection, body weights and tumor sizes of the mice were measured every 3 days, and the tumor volume was calculated using the following equation: tumor volume = (length × width^2^) × 1/2. After 27 days of treatment, the mice were sacrificed, and xenograft tumors were removed for H&E and immunohistochemical staining.

### 2.11 IHC and H&E staining

Formalin-fixed tissue specimens were embedded in paraffin and sliced into serial sections (4 μm thick). Tumor samples were stained with hematoxylin and eosin (H&E). In addition, tumor specimens were immunostained with ki67. Images were captured under a light microscope (Leica, Germany).

### 2.12 ROS measurement

ROS levels were detected based on the oxidation of DCFH-DA by peroxide to produce the fluorescent product 2′,7′-dichlorofluorescein, as previously described. Briefly, treated cells were washed and incubated with DCFH-DA at a final concentration of 10 M for 30 min. After washing, the cells were subjected to flow cytometry (Ex = 488 nm and Em = 530 nm).

### 2.13 MMP detection

The JC-1 assay was performed to determine the MMP [19]. For example, T24 and RT4 cells were treated with carbonyl cyanide *m*-chlorophenyl hydrazone (CCCP, 100 μM; Sigma) for 1 h as a positive control. The cells were harvested and resuspended in PBS containing JC-1 dye (Molecular Probe, T3168) (5 μM) for 30 min at room temperature in the dark. Finally, fluorescence intensity levels were determined by flow cytometry.

### 2.14 ATP measurement

T24 and RT4 cells were treated with Acr (0–100 μM) or CCCP (100 μM; Sigma) for 1 h as a positive control. The cells were harvested and lysed using the cell lysis reagent, and ATP levels in the cell lysates were measured with the ATP bioluminescence assay kit HS II (Roche Molecular Biochemicals, China) using a multimode microplate reader (Tecan, Infinite 200, Austria).

### 2.15 Statistical analysis

One-way analysis of variance was used to analyze experimental data using SPSS version 22.0 software (SPSS Inc., IL, USA). The data are presented as mean standard deviation (SD). A *p* value of less than 0.05 was considered statistically significant (*# means *p* < 0.05, ***means *p* < 0.01).

## 3 Results

### 3.1 MT-12 inhibits proliferation, apoptosis, and autophagy in the BC RT4 and T24 cell lines

**Figure 1:**
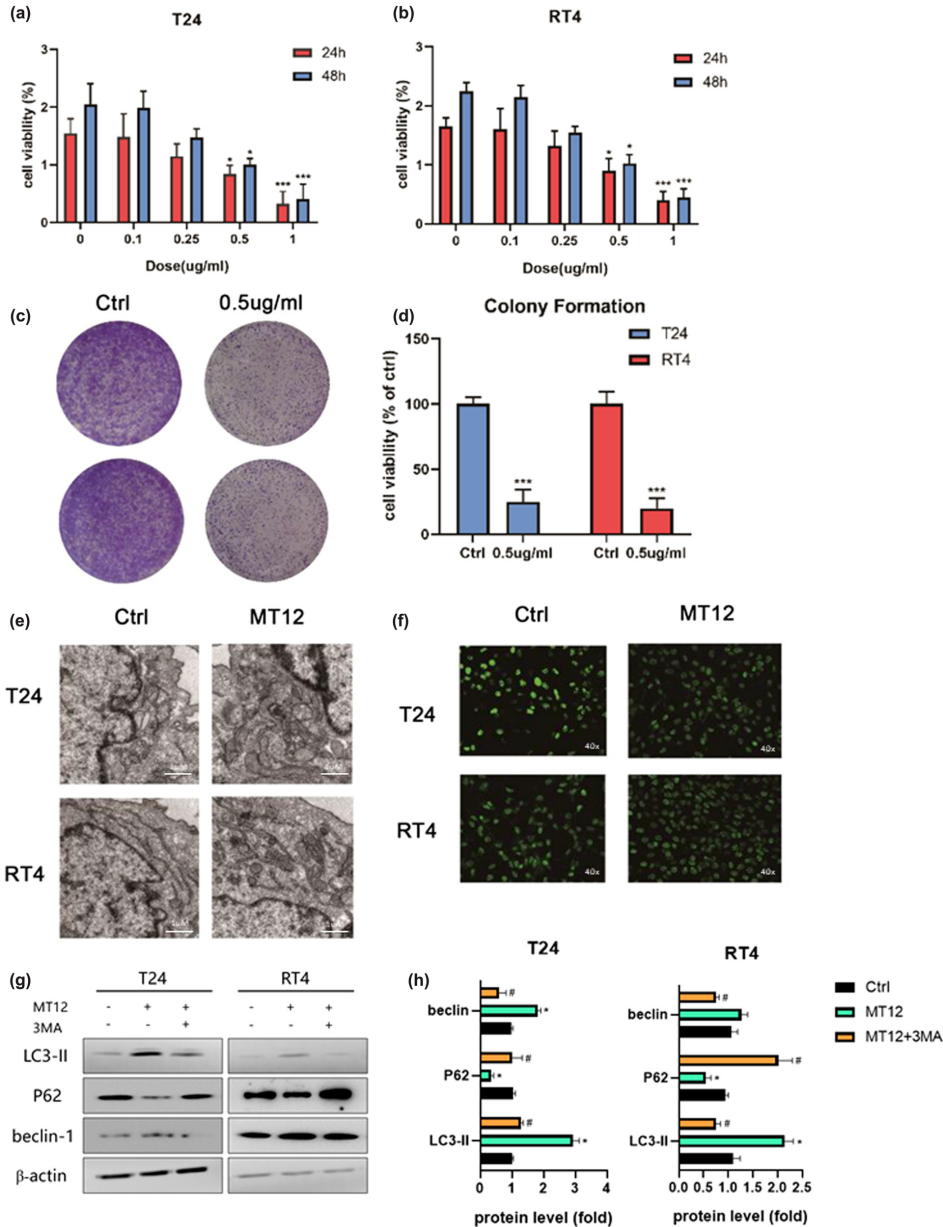
MT-12 inhibits proliferation and autophagy, inducing apoptosis in BC cells. (a and b) In a concentration- and time-dependent manner, MT-12 inhibited the proliferation of BC T24 and RT4 cells. The viability of T24 and RT4 cells was detected by the CCK-8 assay (*n* = 5 per group). (c and d) MT-12 (0.5 μg/mL) inhibited clone formation in T24 and RT4 cells (*n* = 3 per group). (e) The features of apoptosis could be observed in T24 and RT4 cells pretreated with 0.5 g/mL MT-12 by TEM. T24 and RT4 cells treated with MT-12 showed more green fluorescence puncta. (f) GFP-LC3 puncta assays of T24 and RT4 cells after transfection with EGFP-LC3 plasmid in control or MT-12 treated groups. (g and h) The expression of LC3II, P62, and beclin-1 in the control, MT-12, and MT-12 + 3MA groups was detected by Western blot and quantified (*n* = 3 per group). The values are mean SEM. Statistical differences were analyzed by unpaired Student’s *t* test; ^*^ *p* < 0.05; ^#^ *p* < 0.05 (compare with MT12 group).

According to previous results, the IC_50_ of MT-12 in the BC EJ cell line was approximately 0.51–0.66 μg/mL [18]. In this study, BC RT4 and T24 cells were used to confirm the antiproliferative effect of MT-12. The CCK-8 and colony formation assays showed that after treatment with MT-12 (0.1, 0.25, 0.5, and 1.0 μg/mL) for 24 and 48 h, the proliferation of T24 and RT4 cells was suppressed in a time- and concentration-dependent manner (Figure 1a and b). The IC_50_ values of MT-12 in T24 and RT4 cells at 24 h were 0.47 and 0.53 μg/mL, respectively; at 48 h, they were 0.39 and 0.40 μg/mL, respectively. In addition, we also assessed clone formation in T24 and RT4 cells treated with MT-12. The results showed that MT-12 (0.5 µg/mL) inhibited clone formation in bladder RT4 and T24 cells compared with the control group (Figure 1c and d).

Cobra venom cardiotoxins could interact with the outer mitochondrial membrane, and mitochondrial membrane dynamics were found to be associated with autophagy. Thus, autophagy after treatment of BC cells with MT-12 was investigated. TEM results showed that treatment with 0.5 g/mL MT-12 induced the characteristics of apoptosis, including chromatin condensation and margination as well as massive mitochondrial vacuolation, compared with the control group (Figure 1e). Fluorescence electron microscopy showed that more green fluorescence puncta were accumulated in the perinuclear region of MT-12-treated cells compared with the control (Figure 1f). Additionally, to further clarify the autophagy of BC cells after MT12 treatment, we detected the expression of P62 and Beclin-1 in BC cells after MT12 treatment and MT12 + 3MA treatment. The results showed that MT12 significantly downregulated the expression of P62 and significantly upregulated the expression of Beclin-1 and LC3II in BC cells. In the MT12 + 3MA treatment group, compared with the MT12 treatment group, P62 expression was upregulated, while Beclin-1 and LC3II expression was downregulated. These results further demonstrated the autophagy of BC cells after MT12 treatment (Figure 1g and h).

### 3.2 MT-12 induces autophagic cell death in BC cells *in vitro*

Since MT-12 induces significant autophagy and promotes apoptosis in T24 and RT4 cells, we investigated how autophagy contributes to MT-12-mediated inhibition of cell proliferation.

**Figure 2:**
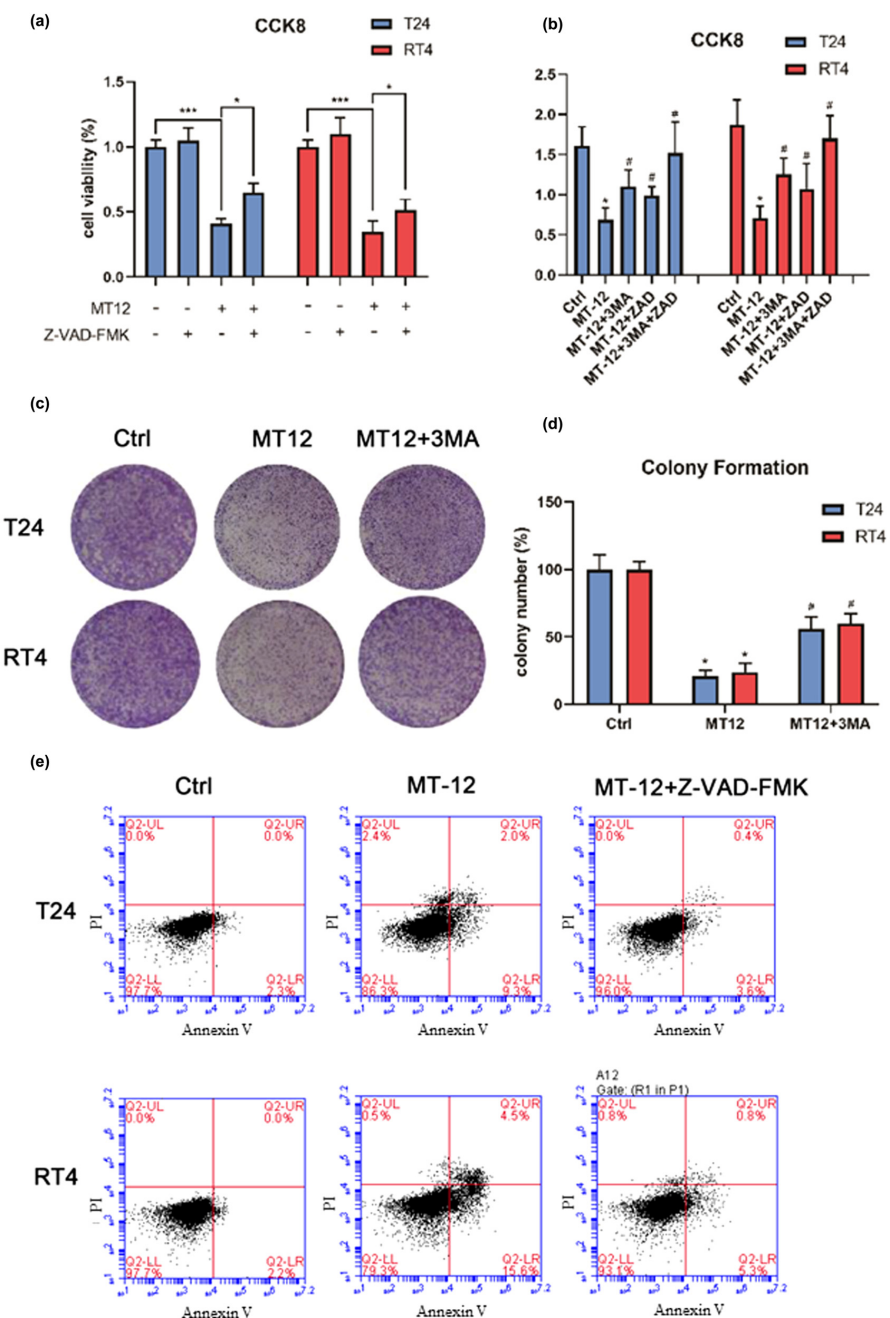
MT-12 induces autophagic cell death in BC cells *in vitro*. (a and b) Cell viability after treatment with the pan-caspase inhibitor Z-VAD-FMK alone or in combination with MT-12 of T24 and RT4 cells was determined by the CCK-8 assay (*n* = 5 per group). (c and d) Clone formation in T24 and RT4 cells administered 3 MA or MT-12 was observed under a microscope (*n* = 3 per group). (e) Apoptosis of T24 and RT4 cells treated with Z-VAD-FMK alone or in combination with MT-12 was detected by flow cytometry (*n* = 3 per group). The values are mean SEM. Statistical differences were analyzed by unpaired Student’s *t* test; ^*^ *p* < 0.05; ^**^ *p* < 0.01.

First, T24 and RT4 cells were treated with the pan-caspase inhibitor Z-VAD-FMK alone or in combination with MT-12 for 24 h. Interestingly, colony formation in BC cells was decreased after MT-12 administration. However, the CCK-8 and colony formation assays showed that the inhibitory effect of MT-12 on BC cells decreased after treatment with Z-VAD-FMK in T24 and RT4 cells, while a suppressive effect persisted (Figure 2a and b).

Autophagy could either be a protective response or lead to cell death. To identify the role of autophagy, we performed the CCK-8 and clone formation assays after administering 3-MA, an autophagy inhibitor, to bladder T24 and RT4 cells. The results showed that 3-MA pretreatment greatly abrogated MT-12-induced cell death (Figure 2c and d).

Next, we determined whether the pan-caspase inhibitor Z-VAD-FMK affected cell death in T24 and RT4 cells. As shown in Figure 2e, Z-VAD-FMK treatment significantly alleviated MT-12-induced apoptosis. These results indicated that MT-12 induced autophagic cell death in BC cells.

### 3.3 MT-12 inhibits the proliferation of T24 tumor xenografts in nude mice

**Figure 3:**
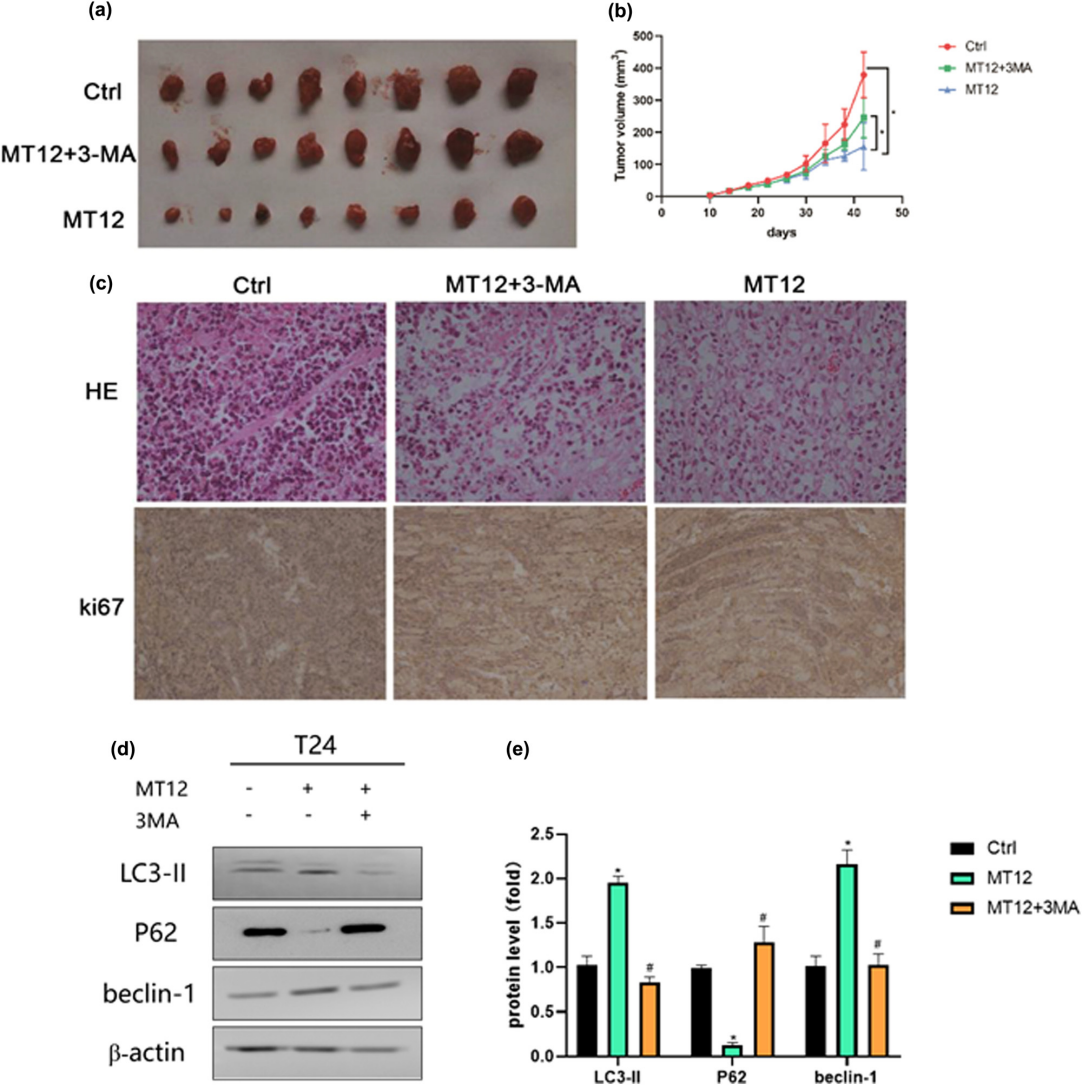
MT-12 induces autophagic cell death in BC cells *in vivo.* (a) Tumors in the subcutaneously transplanted T24 tumor model in nude mice in various groups are shown. (b) Bladder volumes were determined and calculated in various groups. (c) H&E and Ki67 staining were used to assess cell growth in subcutaneously implanted T24 cells. The values are mean SEM. (d and e) The expression of LC3II, P62, in the control, MT-12, and MT-12 + 3MA groups was detected by Western blot and quantified. Statistical differences were analyzed by unpaired Student’s *t* test; ^*^ *p* < 0.05; ^#^ *p* < 0.05 (compare with MT12 group).

A subcutaneously transplanted T24 tumor model in nude mice was established to further confirm MT-12-induced autophagic cell death in BC *in vivo*. As shown in Figure 3a and b, MT-12 inhibited the proliferation of the subcutaneously implanted T24 cells, and 3-MA weakened this suppressive effect. In addition, H&E and Ki67 staining also showed that T24 cell proliferation was significantly suppressed in the MT-12-treated group, while this effect was reversed by 3-MA (Figure 3c). To further clarify the autophagy of BC cells after MT12 treatment, we detected the expression of LC3II, P62, and Beclin-1 in three groups of xenograft model tumors. The results showed that the expression of LC3II and Beclin-1 was significantly upregulated, and the expression of P62 was significantly downregulated in the MT12 treatment group compared with the vehicle control group. After MT12 was combined with posture inhibitor 3-MA, LC3II and Beclin-1 expressions were significantly downregulated, while P62 expression was significantly upregulated compared with the MT12 group. These results further indicated that MT12-induced autophagy and the inhibitory effect of MT12 on cell proliferation was weakened after inhibition of autophagy (Figure 3d and e).

### 3.4 MT-12 induces functional damage in the mitochondria

**Figure 4:**
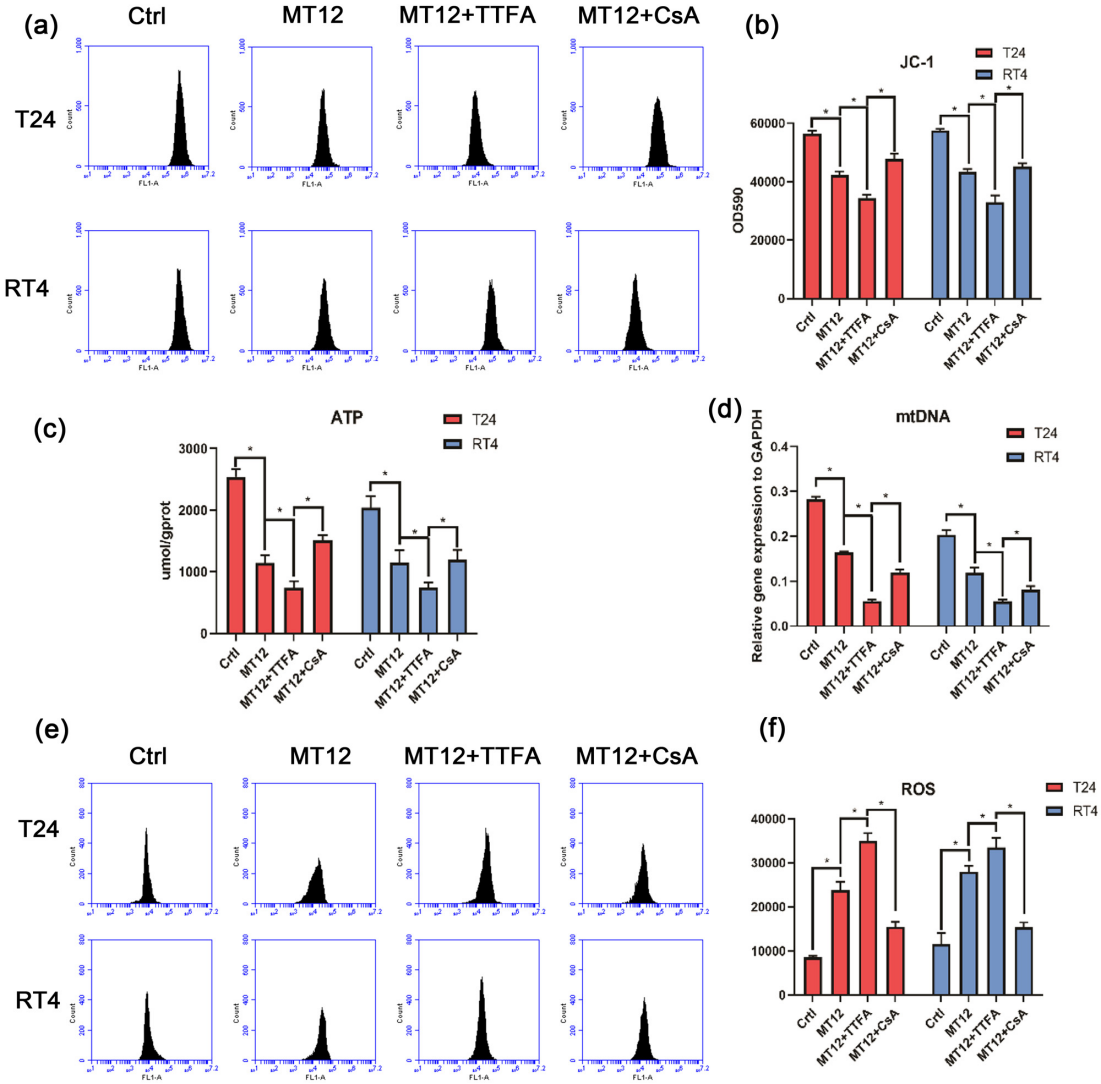
MT-12 induces mitochondrial damage. (a and b) JC-1 was used to assess the MMP, and fluorescence intensity levels were determined by flow cytometry (*n* = 3 per group). (c) ATP amounts in RT4 and T24 cells were determined by colorimetric tests (n = 3 per group). (d) mtDNA expression in RT4 and T24 cells was assessed by qPCR (*n* = 3 per group). (e and f) ROS production in RT4 and T24 cells was determined by flow cytometry (*n* = 3 per group). The values are mean SEM. Statistical differences were analyzed by unpaired Student’s *t* test; ^*^ *p* < 0.05; ^**^ *p* < 0.01.

Previous studies have reported that mitochondrial dysfunction reduces MMP, mtDNA, and ATP production while simultaneously increasing ROS production. According to previous research, mitochondrial dysfunction leads to autophagy in cells. To identify the mitochondrial function in MT-12 treatment, we investigated whether mtDNA, MMP, and ATP were reduced and whether ROS production was significantly upregulated. Thenoyltrifluoroacetone (TTFA), an inhibitor of mitochondrial electron-transport chain complex II, was used to induce mitochondrial damage, and cyclosporine (CsA), an MPTP inhibitor, was used to protect the mitochondria. TTFA or CsA were administered to T24 and RT4 BC cells and then treated with MT-12. We found MMP significantly decreased after treatment with MT-12 alone or combined with TIFA in T24 and RT4 cells, while CsA administration alleviated this effect (Figure 4a and b). Interestingly, mtDNA expression and ATP concentration were obviously reduced after MT-12 treatment and even worsened in the MT-12 and TIFA groups; this inhibitory effect was alleviated by CsA treatment (Figure 4c and d). Moreover, ROS levels were increased after pretreatment with MT-12 alone or combined with TTFA, and ROS amounts were reduced after pretreatment with CsA (Figure 4e and f). These results showed that MT-12 induced BC cell autophagy via mitochondrial damage.

**Figure 5:**
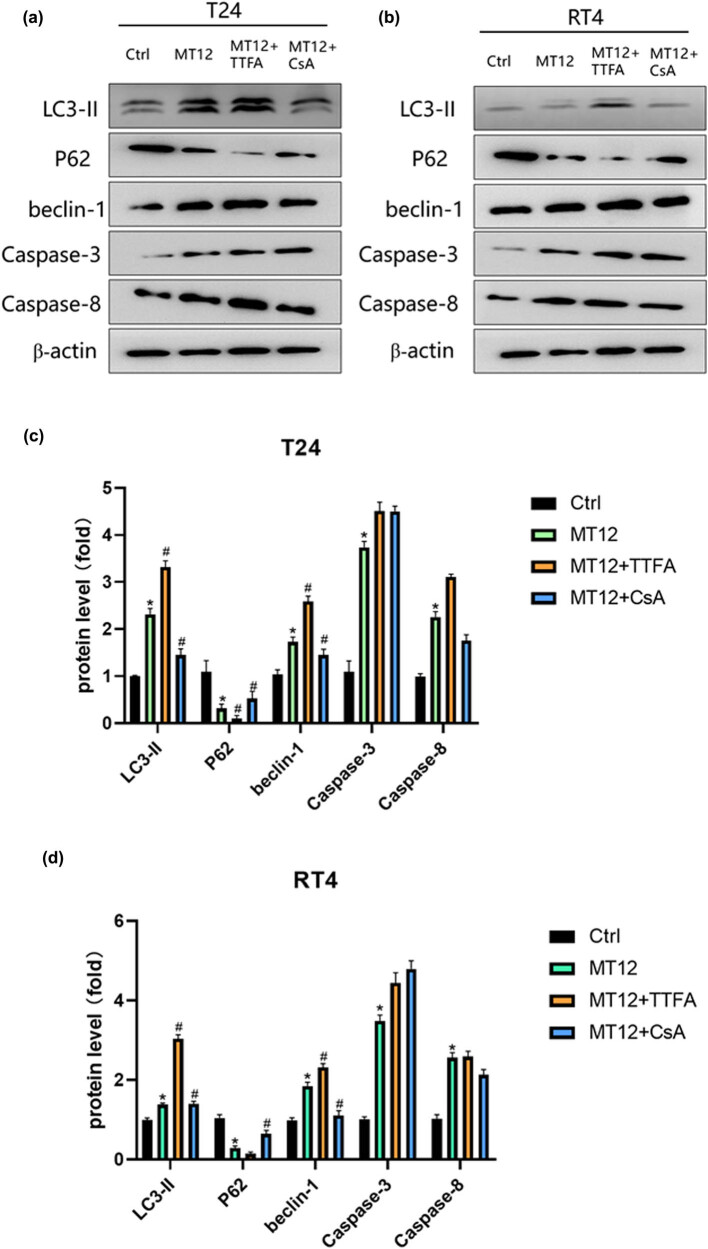
MT-12 induces mitochondrial damage and activates autophagy instead of apoptosis. (a–b) Changes in caspase-3, caspase-8, LC3II, P62, and beclin-1 in T24 and RT4 were determined by Western blot and quantified (*n* = 3 per group). (c–d) The values are mean SEM. Statistical differences were analyzed by unpaired Student’s *t* test; ^*^ *p* < 0.05; ^#^ *p* < 0.05 (compare with MT12 group).

To determine the effect of MT12-induced mitochondrial injury on apoptosis and autophagy of BC cells, we treated them with MT12 monotherapy, MT12 + TTFA, and MT12 + CsA and detected apoptosis-efficient proteins caspase-3 and caspase-8. The expression of LC3II, P62, and Beclin-1 was downregulated by MT12 combined with mitochondrial protective agent CsA on the basis of MT12-induced apoptosis and autophagy in BC cells. The expression of LC3II, P62, and Beclin1 was upregulated, while caspase protein expression was not significantly changed. However, the combination of MT12 and the mitochondrial damage agent TTFA showed the opposite results (Figure 5). These results indicate that MT12-induced mitochondrial damage is more obviously related to autophagy.

### 3.5 MT-12 activates the ROS/JNK/P53 pathway

**Figure 6:**
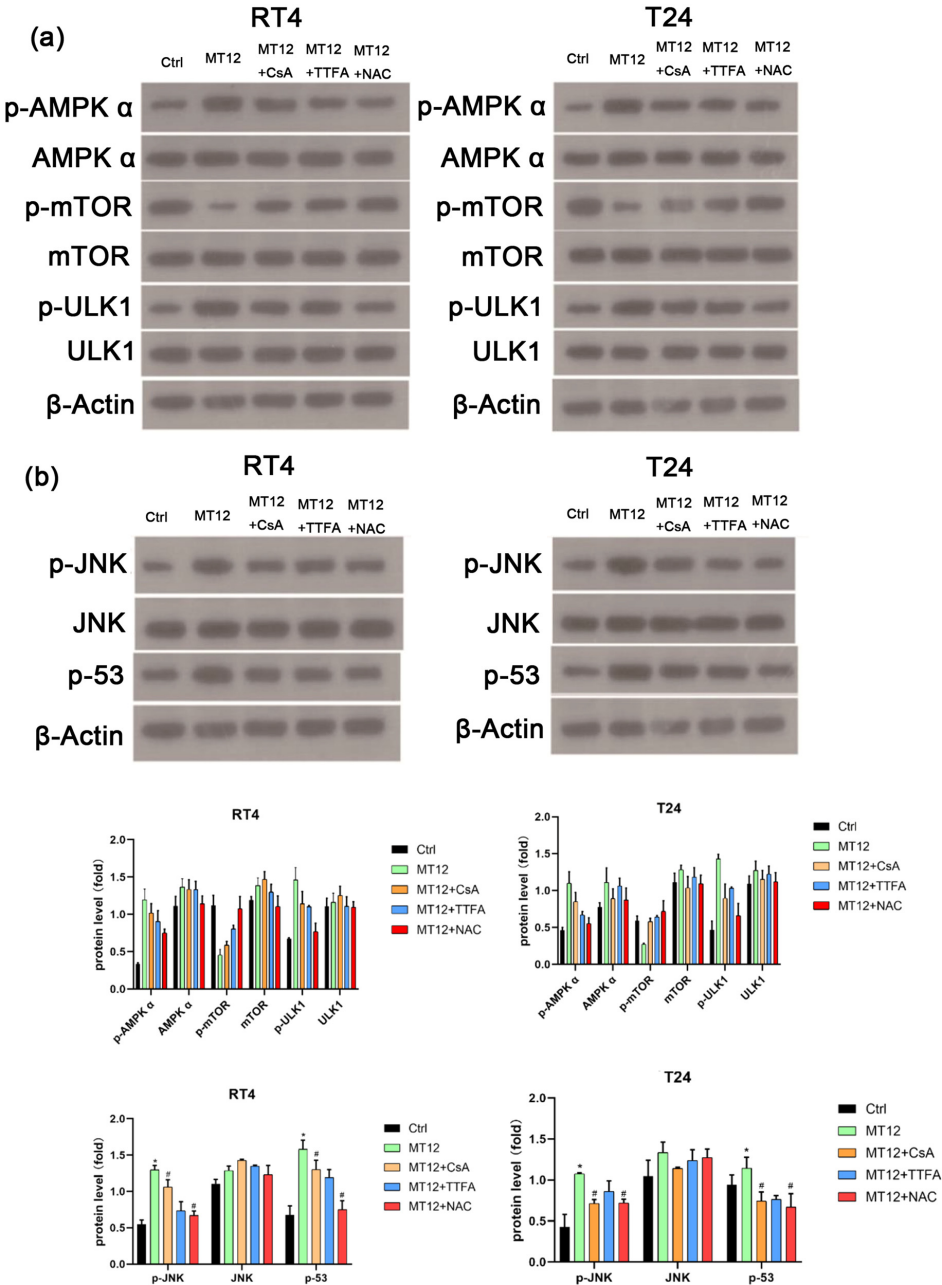
MT-12 activates the ROS/JNK/P53 pathway. (a) Changes in the AMPK/mTOR/ULK1 pathway were determined by Western blot and quantified (*n* = 3 per group). (b) Changes in the p-JNK/p53 pathway were determined by Western blot and quantified (*n* = 3 per group). (c–d) The values are mean SEM. Statistical differences were analyzed by unpaired Student’s *t* test; ^*^ *p* < 0.05; ^#^ *p* < 0.05 (compare with MT12 group).

Recently, studies have reported that ROS production is related to the activation of the AMPK pathway [20]. The AMPK/mTOR/ULK1 pathway is related to cell autophagy. To investigate the potential mechanism, AMPK/mTOR regulation by MT-12 treatment in BC cells was explored. As shown in Figure 6a, after treatment with MT-12, p-AMPK and p-ULK1 were upregulated, and p-mTOR was downregulated; NAC strongly blocked AMPK and ULK1 phosphorylation induced by MT-12. However, CsA and TTFA pretreatments had little effect on the AMPK/mTOR/ULK1 pathway in T24 and RT4 cells after treatment with MT-12. These results suggest that ROS production activates the AMPK/mTOR/ULK1 pathway and induces cell autophagy. However, mitochondrial dysfunction might not be involved in this process.

According to previous studies, the JNK/p53 pathway is related to autophagic apoptosis [21]. To further elucidate the anticancer mechanism of MT-12, we examined the effect of MT-12 on the MAPK-P53 signaling pathway. MT-12 significantly augmented the expression of phosphorylated JNK and P53 (Figure 6b). Furthermore, to explore the associations of mitochondrial damage, ROS accumulation, and ROS/JNK/P53 pathway activation, we investigated p-JNK and p-P53 expression levels following pretreatment with CsA, TTFA, and NAC in T24 and RT4 cells, with subsequent treatment with MT-12. MT-12 induced the expression of p-JNK and p-P53, and TTFA significantly augmented the expression of p-JNK and p-P53 induced by MT-12 (Figure 6b). These results suggest that phosphorylated JNK and P53 were downstream targets in response to mitochondrial damage and ROS accumulation, which could lead to autophagy.

**Figure 7:**
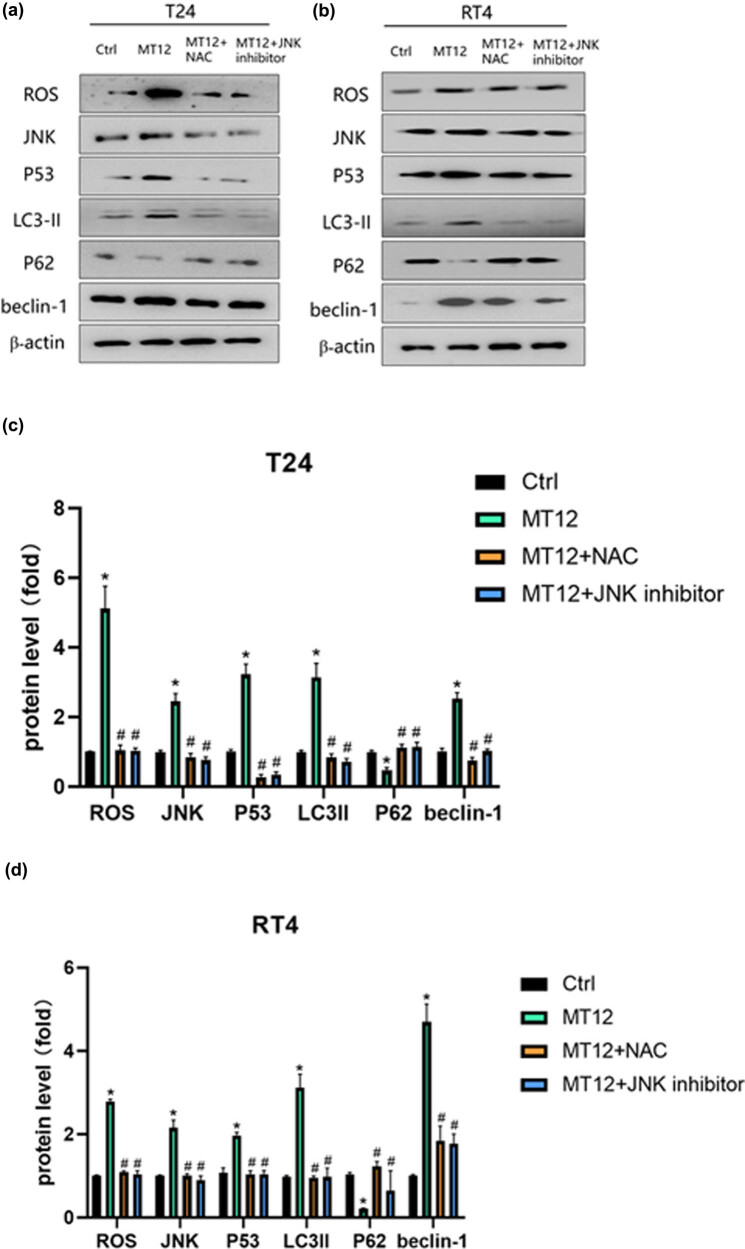
NAC and JNK inhibitor SP600125 block the ROS/JNK/P53 pathway which is activated by MT-12. (a and b) Changes in the p-JNK/p53 pathway and LC3II, P62, and beclin-1 were determined by Western blot and quantified (*n* = 3 per group). (c and d) The values are mean SEM. Statistical differences were analyzed by unpaired Student’s *t* test; ^*^ *p* < 0.05; ^#^ *p* < 0.05 (compare with MT12 group).

We used MT12 monotherapy, MT12 combined with the ROS blocker NAC, and MT12 combined with JNK inhibitors (SP600125) to treat BC cells to see whether it affects autophagy by increasing intracellular ROS and then activating the JNK/P53 signaling pathway. The expression levels of autophagy marker proteins LC3II, P62, and Beclin-1 were detected, and the results showed that mt12-induced changes in autophagy protein expression were reversed regardless of whether the cells were treated with NAC or JNK inhibitors combined with MT12 (Figure 7).

## 4 Discussion

The high recurrence of BC, an increase in grade in patients with recurrence and patients who cannot tolerate the side effects and show resistance to existing chemotherapeutics have been previously reported. This study demonstrated that MT-12 inhibited the proliferation and induced apoptosis of BC *in vitro* and *in vivo* by downregulating autophagy. MT-12 induced mitochondrial damage and decreased autophagy, leading to increased ROS production, activating the JNK/p53 pathway, and causing BC apoptosis. These findings may provide new potential targets for non-muscular-invasive BC in the clinic.

MT is one of the major toxic components of cobra venom and has both cardiotoxic and cytotoxic effects. Its toxic effects on tumor cells play a key role in the study of the antitumor activity of snake venom [22]. Our previous report demonstrated that MT-12 effectively inhibits BC cell growth and metastasis [13], while the mechanism remains unclear. Consistent with previous results, we investigated whether the proliferation of BC cells was inhibited by MT-12 in a concentration- and time-dependent manner. The IC_50_ values of MT-12 in T24 and RT4 cells were 0.39–0.53 g/mL, corroborating our previous study. We also found that Z-VAD-FMK, a pan-caspase inhibitor, weakened the degree of apoptosis after treating BC cells with MT-12. Interestingly, the proliferation of BC cells treated with Z-VAD-FMK and MT-12 in combination was not suppressed. This finding suggests that in addition to apoptosis, another pathway is involved in BC cell inhibition.

Autophagy, now termed type II PCD, is a mechanism by which an organism degrades damaged or misfolded cellular proteins [23]. In terms of biological function, in the event of stress, autophagy prevents the accumulation of toxic or carcinogenic damage to proteins and organelles to inhibit cell transformation; on the other hand, once tumors form, autophagy provides more abundant nutrients to cancer cells, thereby promoting tumor growth. Briefly, autophagy is considered a “double-edged sword” in the process of tumor occurrence and development [24]. Our results demonstrated that autophagy and apoptosis were inhibited by 3-MA and Z-VAD-FMK administered separately or together, and the viability of BC cells was recovered after treatment with MT-12, which suggested an antitumor role by inducing autophagy in BC.

LC3 is involved in the formation of autophagosomes. High expression of LC3, especially LC3II, is closely related to the proliferation, invasion, and metastasis of solid tumors. The expression of LC3II is proportional to the degree of autophagy [25,26]. For example, findings by Sato et al. [27] showed that 90% of colon cancers and 100% of all lymph node and liver metastases had LC3 expression. Our results demonstrated increased LC3 green fluorescence puncta in T24 and RT4 BC cells. These results indicate autophagy contributes to the mechanism of MT-12 in T24 and RT4 cells.

Additionally, the opposite effect of MT-12-induced autophagy has been observed *in vivo*. In nude mice, T24 cells formed tumors subcutaneously, and MT-12 significantly inhibited tumor growth. H&E staining and immunohistochemical results showed that MT-12 inhibited cell growth in subcutaneously implanted T24 cells and downregulated Ki67. These effects were reversed by 3-MA. Ki67, an important indicator of tumor cell activity, is closely associated with the development and progression of tumor cells [28]. MT-12 decreased the expression of ki67, indicating that MT-12 inhibits the development and progression of BC cells.

MT-12 is an obvious polar amphoteric molecule. This amphoteric structure is beneficial for the binding of MT-12 to the cell membrane. The mitochondria, the centers of energy metabolism, have a bilayer membrane structure [29]. We assessed whether MT-12 also acts on the mitochondrial bilayer membrane, similar to its effect on the cell membrane. Therefore, we evaluated the mitochondrial function. In this study, treatment of BC T24 and RT4 cells with MT-12 resulted in mitochondrial dysfunction, including decreased MMP, mtDNA, and ATP, and increased ROS, compared with NC. The above results further confirmed the role of the mitochondria in the apoptotic effect of MT-12.

ROS accumulation promotes mitochondrial damage, and mitochondrial dysfunction induces ROS production, which is a vicious cycle. Studies have reported that increased ROS and mitochondrial damage induce autophagic death in cervical cancer cells due to ionizing radiation [30]. In addition, chamomile induces caspase-independent autophagic death by activating ROS in osteosarcoma cells [31]. Therefore, we believe that MT-12 induces autophagy in BC cell lines via the mitochondrial pathway. The regulation of autophagy is closely related to autophagy-related signaling, including mTOR-dependent (AMPK/mTOR and PI3K/Akt/mTOR) and non-mTOR-dependent (p53) pathways. In this study, after MT-12 treatment, p-AMPK, p-JNK, p-ULK1, and p-p53 were upregulated, and mTOR was downregulated. Phosphorylation of JNK and p53 was strongly blocked by CsA and NAC pretreatment, and TTFA significantly enhanced MT-12-induced expression of p-JNK and p-p53. These results suggest that phosphorylated JNK and p53 are downstream targets in response to mitochondrial damage and ROS accumulation, which leads to autophagy. MT-12 strongly blocked AMPK and ULK1 phosphorylation in BC cells pretreated with NAC. However, the AMPK/mTOR/ULK1 pathway in T24 and RT4 cells after MT-12 treatment was not significantly affected by CsA and TTFA pretreatment. These results suggest that ROS production activates AMPK via the AMPK/mTOR/ULK1 pathway to induce autophagy. However, mitochondrial dysfunction may not be associated with this process.

In conclusion, our results further explained the mechanism of MT-12 that decreased autophagy and induced mitochondrial damage in BC. In contrast to our previous study, we found autophagy plays a vital role in MT-12-induced BC apoptosis, which may provide more possible targets for BC clinic treatment.

## Figures and Tables

**Figure A1:**
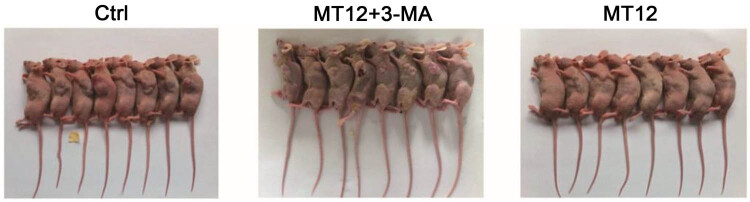
The growth of t24 cells in subcutaneously implanted tumours in Ctrl, MT12+3-MA and MT-12 treatment group.
